# *International Journal of Environmental Research and Public Health* Best Paper Award 2013

**DOI:** 10.3390/ijerph10010443

**Published:** 2013-01-23

**Authors:** Paul B. Tchounwou, Ophelia Han

**Affiliations:** 1 Molecular Toxicology Research Laboratory, Jackson State University, 1400 Lynch Street, Box 18750, Jackson, MS 39217, USA; E-Mail: paul.b.tchounwou@jsums.edu; 2 MDPI AG, Postfach, CH-4005 Basel, Switzerland and MDPI Branch Office, Beijing, China; E-Mail: ophelia.han@mdpi.com

With the start of 2013, *International Journal of Environmental Research and Public Health* is instituting an annual award to recognize outstanding papers related to environmental health sciences and public health that meet the aims, scope and high standards of this journal. We are pleased to announce the first “*International Journal of Environmental Research and Public Health* Best Paper Award” for 2013. Nominations were solicited from the Editorial Board members, with all papers published in 2009 eligible for consideration. The following five papers were awarded:

## Article Award:

### 1^st^ Prize

**Authors: Patricia A. Richter, Ellen E. Bishop, Jiantong Wang and Monica H. Swahn**

Article title: Tobacco Smoke Exposure and Levels of Urinary Metals in the U.S. Youth and Adult Population: The National Health and Nutrition Examination Survey (NHANES) 1999–2004

*Int. J. Environ. Res. Public Health ***2009**, *6*(7), 1930–1946; doi:10.3390/ijerph6071930

Available online: http://www.mdpi.com/1660-4601/6/7/1930

### 2^nd^ Prize

**Authors: Athanasios Valavanidis, Thomais Vlachogianni and Konstantinos Fiotakis**

Article title: Tobacco Smoke: Involvement of Reactive Oxygen Species and Stable Free Radicals in Mechanisms of Oxidative Damage, Carcinogenesis and Synergistic Effects with Other Respirable Particles

*Int. J. Environ. Res. Public Health ***2009**, *6*(2), 445–462; doi:10.3390/ijerph6020445

Available online: http://www.mdpi.com/1660-4601/6/2/445

### 3^rd^ Prize

**Author: Melanie Swan**

Article title: Emerging Patient-Driven Health Care Models: An Examination of Health Social Networks, Consumer Personalized Medicine and Quantified Self-Tracking

*Int. J. Environ. Res. Public Health ***2009**, *6*(2), 492–525; doi:10.3390/ijerph6020492

Available online: http://www.mdpi.com/1660-4601/6/2/492

## Review Award:

### 1^st^ Prize

**Authors: Jong-Su Seo, Young-Soo Keum and Qing X. Li**

Article title: Bacterial Degradation of Aromatic Compounds

*Int. J. Environ. Res. Public Health ***2009**, *6*(1), 278–309; doi:10.3390/ijerph6010278

Available online: http://www.mdpi.com/1660-4601/6/1/278

### 2^nd^ Prize

**Authors: Johanna Brinkel, Mobarak H. Khan and Alexander Kraemer**

Article title: A Systematic Review of Arsenic Exposure and Its Social and Mental Health Effects with Special Reference to Bangladesh

*Int. J. Environ. Res. Public Health ***2009**, *6*(5), 1609–1619; doi:10.3390/ijerph6051609

Available online: http://www.mdpi.com/1660-4601/6/5/1609

The prize awarding committee merits the article “Tobacco Smoke Exposure and Levels of Urinary Metals in the US Youth and Adult Population: The National Health and Nutrition Examination Survey (NHANES) 1999-2004” as “…a nice epidemiological study that establishes a correlation between tobacco smoke and heavy metals exposure. It further provides a comprehensive analysis of the link between exposure and health disparities…”. The article “Tobacco Smoke: Involvement of Reactive Oxygen Species and Stable Free Radicals in Mechanisms of Oxidative Damage, Carcinogenesis and Synergistic Effects with Other Respirable Particles” reported “…an excellent research that elucidates the role of oxidative stress on tobacco-smoke-induced toxicity and carcinogenicity. It further demonstrates its synergistic effects with other respirable particulate matters…”. The article “Emerging Patient-Driven Health Care Models: An Examination of Health Social Networks, Consumer Personalized Medicine and Quantified Self-Tracking” discussed “…novel strategies to health care delivery by examining social networks, personalized medicine, and quantified self-tracking…”.

These five exceptional papers are valuable contributions to the *International Journal of Environmental Research and Public Health* and the public health field. On behalf of the Prize Awarding Committee and the Editorial Board of *International Journal of Environmental Research and Public Health*, we would like to congratulate these five teams for their excellent work. In recognition of their accomplishment, Drs. Patricia A. Richter, Athanasios Valavanidis and Melanie Swan will receive a prize of 600 CHF, 400 CHF, and 200 CHF, respectively, and the privilege to publish an additional paper free of charge in open access format in the *International Journal of Environmental Research and Public Health*, after normal peer-review. Drs. Qing X. Li and Mobarak H. Khan will be awarded the privilege of publishing an additional research paper free of charge in open access format in the *International Journal of Environmental Research and Public Health*, after normal peer-review. 

## Prize Awarding Committee

*Editor-in-Chief*
**Prof. Dr. Paul B. Tchounwou**
  Molecular Toxicology Research Laboratory, Jackson State University, 1400 Lynch Street, Box 18750, Jackson, Mississippi 39217, USA
  E-Mail: paul.b.tchounwou@jsums.edu
*Editor-in-Chief,* Section ‘Health Economics’
**Prof. Dr. Ulf-G. Gerdtham**
  Department of Clinical Sciences, Department of Economics, Lund University, P.O. Box 7082, S-220 07 Lund, Sweden
  E-Mail: ulf.gerdtham@nek.lu.se
*Editorial Board Member and Guest Editor*
**Dr. Zubair Kabir**
  Department of Epidemiology & Public Health, University College Cork, Cork, Ireland
  E-Mail: z.kabir@ucc.ie
*Managing Editor*
**Dr. Ophelia Han **
  MDPI Beijing Office, Suite 2011, Ruidu International Center, No. 1 Cuijingbeili, Tongzhou District, Beijing, China
  E-Mail: ophelia.han@mdpi.com

